# Spatio–Environmental Analysis of *Vespula germanica* Nest Records Explains Slow Invasion in South Africa

**DOI:** 10.3390/insects12080732

**Published:** 2021-08-16

**Authors:** Ruan Veldtman, Derek Daly, Gerard F. H. v. G. Bekker

**Affiliations:** 1 South African National Biodiversity Institute, Kirstenbosch Research Centre, Private Bag X7, Claremont 7735, South Africa; drderekdaly@yahoo.co.uk; 2 Conservation Ecology and Entomology, Stellenbosch University, Private Bag X1, Matieland 7602, South Africa; gulu@sun.ac.za

**Keywords:** social wasps, Mediterranean climate, moisture stress, optimised hot spot analysis

## Abstract

**Simple Summary:**

Social wasp invasions can spread quickly and have serious impacts if they reach new regions with favourable climatic conditions. However, in areas less suitable to them, invasion patterns can show factors that may prevent their spread. We use nest records of the German wasp from the southern tip of South Africa, to map and analyse what habitats they seem to prefer. Factors investigated included temperature, rainfall, and moisture availability. We find that this invasive wasp prefers moister and cooler conditions, and because these habitats are patchy in the region they have invaded in South Africa, they can only spread slowly unassisted, and utmost between 50 and 200 km with the assistance of humans. This is the likely reason for the very slow invasion seen in South Africa. The spatial patterns we quantify here make it possible to use a remote-sensing approach to determine the suitability of an area for future invasions. Predicting the likelihood of invasions will greatly aid management actions. Public awareness around the potential accidental transport of the German wasp and similar species should also be undertaken.

**Abstract:**

Investigating the distributions of invasive species in marginal habitats can give clues to the factors constraining invasive spread. *Vespula germanica* is the most widely distributed of all the invasive Vespids, which in the Southern Hemisphere typically have large extensive invasive populations. In contrast, the invasion into South Africa has been slow and is still confined to a small geographic area. Here we analyse the distribution of all recent nest records in South Africa (n = 405). The distance to main rivers, mean annual rainfall, summer normalised difference moisture index (NDMI) values, and mean annual temperatures (average, minimum, maximum, and summer maximum temperature) was measured for every nest. We find that value ranges of these variables are different between the value ranges recorded for nests, the general distribution area of the wasp, and the area of absence. Optimised Hot Spot Analysis was used to quantify spatial structure in the measured climatic variables. Generally, factors related to moisture stress set the environmental limits of *V. germanica*’s landscape distribution. Due to the strong preference of nesting sites close to river courses, for higher rainfall conditions, medium to medium-high NDMI values, and lower mean annual temperatures, it is unlikely that *V. germanica* will be able to spread uniformly where it is currently found in South Africa.

## 1. Introduction

Defining the environmental conditions that promote the diffusion of invasive species in newly introduced areas is a key objective in invasion biology [1,2,3]. After invasive species establish, it is important to know potential areas of future spread and how large populations are likely to become. For species that are highly successful invaders, colonization and spread can be rapid, making it difficult to sequentially map invasive spread. In contrast, invasive species that are transported to marginal habitats (relative to their climatic niche in their native range; e.g., [4]) will likely spread slowly and the analysis of their distribution can give useful information on limiting factors. Although the rate of spread and population densities under marginal conditions are much lower than in climatically optimal ranges, any established marginal populations could act as stepping-stones for future invasions into more suitable areas (e.g., [5]). Furthermore, due to increasing global trade and travel, the likelihood of future accidental introductions of invasive species into urban centres is increasing [6,7]. A detailed understanding of such invasions in marginal environments is thus important.

Distribution modelling of invasive species can be conducted to forecast regions that are likely to be invaded after successful establishment. For example, distribution modelling programs such as MAXENT or CLIMEX can take into account the importance of multiple climatic and environmental factors, resulting in composite habitat suitability predictions [8,9,10,11,12]. However, such modelling is not useful at smaller than regional or national scales when there are relatively small environmental gradients, which is often the extent of invasive species recently detected in marginal habitat. Furthermore, models resulting in a single predicted suitability index potentially obscures the ability of invasive species to overcome unsuitable conditions at local scales. For example, invasive species limited by hot and dry conditions can survive if they occur near water sources (e.g., [13]), or survive in colder environments by selecting warmer urban areas [14].

Globally, invasive social wasps rank among some of the worst invasive species [15] with several examples of rapid invasive spread [16,17,18,19]. Apart from the severe ecological damage caused [20,21], social wasp invasions can have significant implications for municipal utilities and conservancy boards where they occur [16,22]. For example, in Australia and New Zealand removal of invasive wasp nests in urban and semi-urban areas represents the use of significant monetary and human resources [21,22,23]. It is likely that the pattern of invasion of these wasps in highly suitable vs. marginal areas are in stark contrast and are operated by different mechanisms [19,24,25].

The German wasp or yellowjacket, *Vespula germanica* (Fabricius, 1793) (Hymenoptera: Vespidae), is the most widespread Vespid wasp in the world [20,21,26]. It is native to Europe, the Mediterranean and Asia where it is an important insect predator and scavenger [27]. In its invaded range in the southern hemisphere, population densities can be very large due to highly favourable habitat conditions experienced before winter die back occurs (e.g., Southern Argentina; [19,28]), or in the absence of winter dieback, colonies become perennial and can grow much larger than in their native range [13]. Moist temperate regions with abundant food resources (honeydew and invertebrates) such as in Argentina, New Zealand and Tasmania invasive wasp biomass can reach epidemic proportions [19,29]. Under such conditions this invasive species has severe ecological and socio-economic impacts [21,29,30].

Moisture stress and wet heat stress (humid, tropical conditions) have been shown to be the two most significant limits on *V. germanica*’s global distribution [11,31]. Furthermore, distribution records of *V. germanica* in more climatically marginal regions of Argentina (see also [24]) and Australia were better explained if irrigation was added to the existing moisture subsidy (i.e., mean annual precipitation) of locality records [11]. Kasper et al. [32] found that between 20 °C and 35 °C foraging rates begin to reduce to 50% of the daily maximum, above 35 °C foraging rate drops rapidly, and stops completely at 40 °C. Additionally, under hot conditions foragers thermoregulate their body temperature by regurgitating water and use water in evaporative cooling to keep nests at optimum temperatures [32]. High temperatures thus increase the need for water in this species and populations may be severely impacted in seasonally hot regions where water is limited. Horwood et al. [13] previously suggested that water availability was a key factor in limiting *V. germanica*’s distribution in the Sydney Metropolitan area. The distribution of *V. germanica* in Australia and other parts of their introduced range might be limited to areas experiencing cooler temperatures, and to places with nearby water. In summary, it appears that *V. germanica* can persist in regions with marginal climatic conditions, provided there is water supplementation by humans or river courses in close proximity.

*Vespula germanica* is listed as a category 1B invasive species in South Africa which by law must have an invasive species management plan [33]. Compared to other invaded regions [16,17,34,35,36,37] the invasion of *V. germanica* in South Africa has been unusually slow and it is still geographically limited [33,38,39]. Since it was first recorded in Cape Town in 1972 [40], the species has not moved outside of the south-western tip of the Western Cape. It currently occurs in a general region of intermediate climatic suitability despite large climatically suitable areas to the east [35], while less suitable regions can also be occupied if improved by irrigation [11]. Therefore, the question arises: why after almost 50 years is only such a small part of South Africa invaded? This is despite the most recent CLIMEX modelling indicating that all previous records of *V. germanica* in South Africa are surrounded by climatically optimal and highly suitable habitat (i.e., [11]). Here we use all available nest records (except historic records) to analyse patterns and possible factors that can explain this constrained invasion. Our aim was to describe and test for any spatial structure in those factors that are likely to significantly constrain the distribution of *V. germanica* in South Africa. This works also serves as a detailed case study to understand the spread of an invasive species under more marginal conditions.

## 2. Materials and Methods

### 2.1. Search for V. germanica Nests and Georeferencing

All *V. germanica* nests reported between 2012 to 2019 by the public, alien and invasive species control teams, beekeepers, farmers, and found during our own field surveys (58%) were recorded and georeferenced. Prior to this period, few accurate georeferenced records are available [41] and historical records were consequently not included in the data. At the start of 2012, after the prolific spread of the European paper wasp (*Polistes dominula*) in the Northern suburbs of the Cape Metropolitan [38], the South African National Biodiversity Institute (SANBI)—Invasive Wasp Project was launched including a public awareness campaign from SANBI, Stellenbosch University, and the City of Cape Town. This included identification pamphlets that were distributed by postgraduate students, while local newspapers ran several reports on the wasp invasion. The Invasive Species department of the City of Cape Town also created a website sighting-tracker for residents to report wasp problems.

The Cape Metropolitan area that encompasses most of all urban areas in the study region, has a high human population density, thus increasing the probability of finding nests. In turn, in the surrounding rural areas, there are farms or other tourist attractions which given the wasp’s propensity for being a pest, the chances are good of nests or at least sightings being reported. We thus assume that in the urban areas and the surrounding rural areas respectively in the study region, the respective ‘search effort’ for *V. germanica* is uniform within each of these two contexts. However, the greatest proportion of nest records were found by our own active searches of landscapes and municipal areas, targeting gaps from previous years, which did include the full gradient of landscape environmental conditions. Consequently, the specific year in which a nest was recorded was not analysed because active searches did not expend equal effort across the entire study period.

**Figure 1:**
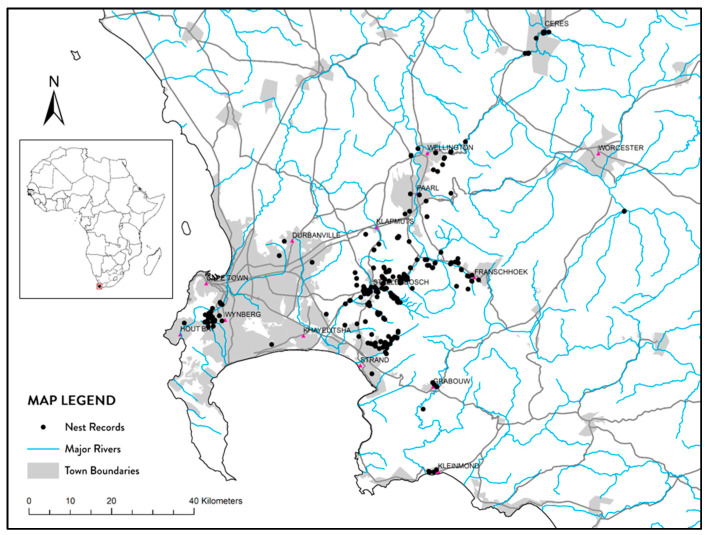
Map showing the distribution of *V. germanica* nest records in the Western Cape relative to indicators of human activity (i.e., town municipal areas and major road networks) and major river courses.

All nest location data (x, y coordinates), as well as spatial attribute data were incorporated into a Geographic Information System (GIS), in order, to manage, explore, analyse and present nest occurrence data spatially (Figure 1). The environmental variable data used included long-term (30 years) annual means for rainfall, temperature, minimum temperature, maximum temperature, and summer maximum temperature (maximum temperatures from January to March) (incorporated and/or extracted within the GIS are listed in Table S1). In addition, the Normalised Difference Moisture (Water) Index (NDMI/NDWI) instead of Normalised Difference Vegetation Index (NDVI) was used to quantify landscape moisture availability. The latter is more influenced by the reflection of radiation from surfaces than NDMI values, which more directly characterises above ground moisture availability/absorption and are commonly used in agricultural applications (e.g., [42]). We used average NDMI values measured during the peak moisture stress period within our study area (i.e., January to March). High NDMI values (values approaching 1) are indicative of high moisture availability; while low NDMI values (values approaching −1) indicate low moisture availability (e.g., heavily vegetated areas will have the highest values and un-vegetated sandy areas the lowest). All attributes listed in Table 1 were determined for each nest location using ArcGIS 10.6.1.

### 2.2. Comparison of Selected versus Available Environmental Conditions

To compare the environmental conditions where *V. germanica* is found relative to where it is absent, a buffer radius of 2.5 km was created around each recorded nest to approximate the area of occurrence (Figure S1). Queens can naturally spread approximately 1 km per year in their invasive range [18], thus using a larger buffer radius we can account for any natural spread in the immediate local area within a few years before or after a given nest record. The entire buffered area was then descriptively analysed (mean and standard deviation [SD]) as a single polygon for all environmental variables using ArcGIS 10.6.1. Nest records for which there was multiple topographical barriers (i.e., mountain ranges) from the more continuous distribution were excluded (20 nests). This was done to remove potential bias in calculating average encountered conditions in the area of absence and occurrence. All quarter degree cells (15′ by 15′) that contained parts of the buffered distribution were selected as well as unoccupied cells adjacent to the cells containing the initial invasion point (i.e., Kirstenbosch, Cape Town) up until the same distance in all compass directions. This process resulted in a 3 × 4 quarter degree grid being selected as the maximum extent of the potential distribution range (i.e., if wasps could spread at the same rate in all directions from the original point of introduction) (Figure S1). Thus, the area of *V. germanica* absence was calculated as the full terrestrial extent of the distribution minus the area of occurrence.

For every 10 × 10 m cell found within the area of extent, values for every environmental variable were measured. For comparison, values for every sampled nest (for the same variables) were also recorded. Due to the different types of data (area data versus point data) and amount of data (mean and SD calculated from >9 million measurements versus n < 500) no statistical tests could be done to determine statistically significant differences. Instead, descriptive statistics (mean and SD) was used to compare the value distributions of environmental variables between the area of absence, the area of occurrence and that of the actual nest records. If there are no differences, it indicates random selection of nesting sites relative to the existing environmental conditions found where the wasp is absent or present, respectively. If there are differences, it could indicate the direction of possible nesting preferences of the wasp.

### 2.3. Analysis of Spatial Structure in Environmental Variables

Optimised Hot Spot Analysis (OHA) was used to statistically describe spatial structure in the values of the environmental variables measured for nest records, using ArcGIS 10.6.1. This method uses the Getis–Ord Gi* statistic [43] to characterise the intensity of the clustering of high values (hot spots) or low values (cold spots) in the data without the use of traditional statistical tests (e.g., [44,45]). Spatial autocorrelation, using the Global Moron’s I statistic, is calculated at incrementing distances in order to determine the scale of analysis (neighbourhood) (e.g., see Jossart et al. [46]), after which the Getis–Ord Gi* statistic is used to determine whether the clustering of high and low values based on the neighbourhood are statistically significant (i.e., it assesses each feature within the context of its neighbouring features and then compares the derived local statistic to the global statistic) [43]. The results from the Getis–Ord Gi* statistic are automatically corrected for multiple testing and spatial dependence by making use of the false discovery rate correction method. The OHA tool classified each nest record, based on the attribute tested as either a statistically significant hot spot or cold spot or as non-significant. Statistically significant nests were binned into three groups that reflects hot- and cold spots at a 99, 95 and 90% confidence level, while all non-significant nests were binned to show the non-significant spots (i.e., seven discrete groups in total). Maps of hot and cold spots were created for visual analyses using ArcGIS.

## 3. Results

**Figure 2:**
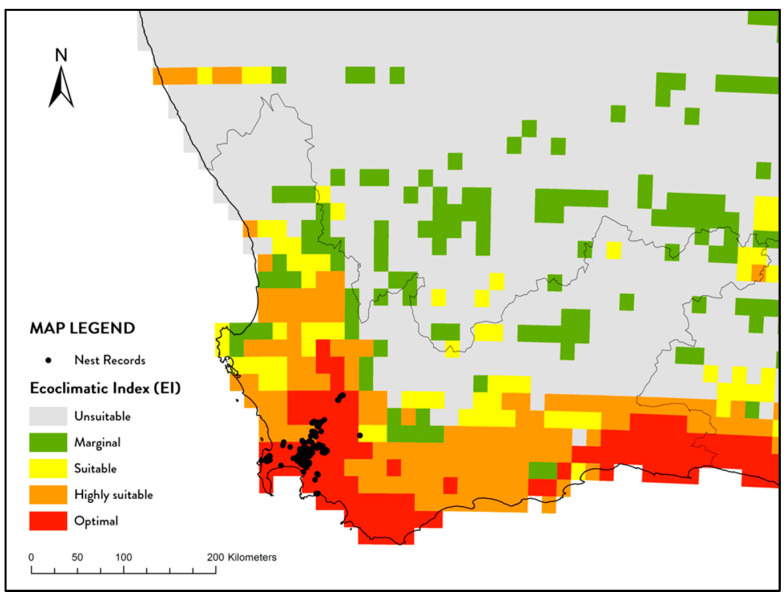
Distribution of V. germanica nests recorded in South Africa (2012 to 2019) and CLIMEX overlay indicating climatic scheme [11].

A total of 405 nests were recorded between 2012 and 2019 (with respectively 2, 10, 64, 8, 105, 63, 109, and 44 nests recorded per year). Most nests (certainly the main clusters) are in the shadow or proximity of mountains. There was a negative association between nest records and surrogates of human activity: urban areas typically had fewer nest records and although some nests were in close proximity to roads (especially in areas of low nest density) aggregations of nests did not show the same association with major roadways (Figure 1). Overlaying published CLIMEX climatic modelling layer for South Africa it is evident that all recorded nests occur in either optimal or highly suitable climatic zones (Figure 2). However, it is clear that despite much of this region being predicted as suitable for *V. germanica*’s occurrence, the realized distribution is still very small. The value ranges of environmental variables, measured for nest records, are shown in Table 1.

### 3.1. Comparison of Selected versus Available Environmental Conditions

**Figure 3:**
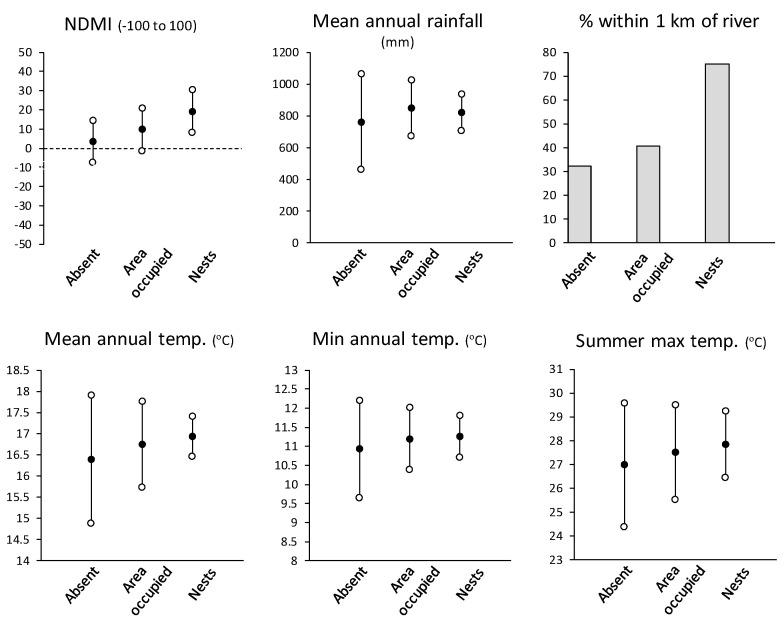
Comparison of means ± standard deviations for studied environmental variables between *V. germanica*’s area of absence and area of occurrence and actual nest records. Distance to main rivers is a relative measurement and was instead expressed as the percentage of the area within 12 selected quarter degree grids (see methods; Figure S1), or percentage of nests, within 1 km of a main river.

There were marked differences in the range of values (mean and standard deviations) for all analysed environmental variables between the area where *V. germanica* was absent, its area of occurrence and between the actual nest records (Figure 3). First, the frequency distribution of NDMI values of nest records were higher than in the area of occurrence and much higher than the area of absence (Figure 3). This would indicate that *V. germanica* nests are more likely to occur where above average soil moisture was available. It is also noteworthy that the mean NDMI of the area of absence fell outside the normal distribution (mean plus and minus one standard deviation) of the nest records indicating strong selection for higher-than-average NDMI values. Mean annual rainfall (MAR) was lower in the area of absence than *V. germanica*’s area of occurrence and for nest records. However, the standard deviations of the MAR of nest record values were narrower compared to the areas of absence and occurrence. This indicates that few nests were found at sites with very high, or especially, very low rainfall.

**Figure 4:**
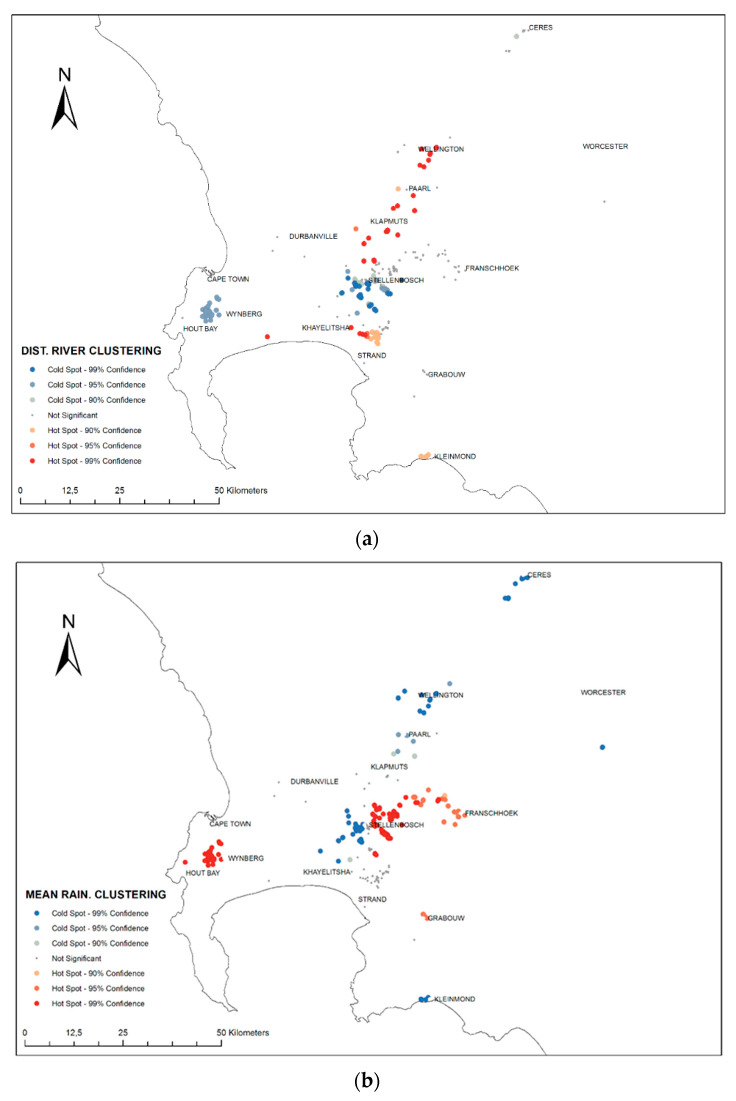
Optimised Hot Spot Analysis maps of (**a**) ‘distance to a main river’, and (**b**) mean annual rainfall for all sampled *V. germanica* nests. A hotspot (red) means significant clustering of high values of the analysed environmental variable, while a cold spot (blue) shows significant clustering of low values (and not clustering of the nest records themselves).

With the distance to main river data, a bigger percentage of the area of occurrence was within 1 km of a river compared to the area of absence, although the percentage of nests within 1 km of a river, was almost double than that of the area of occurrence. There is thus strong selection for nests in closer proximity to main rivers compared to average available conditions. For all temperature variables, means were higher in areas where *V. germanica* was present than where it was absent (by 0.25 to 0.75 °C) but the normal range of conditions (mean ± on SD) were less variable by 1.0 and 2.0 °C in the area of occurrence and for nest records, respectively (Figure 4). This indicates that nesting sites excluded temperature extremes compared to the average available temperatures. Drier areas typically have far more temperature variation than areas with more moisture such as vegetated areas. It is thus likely that all the temperature variables are also influenced by moisture availability. Due to topography, mountain tops in the study area receive high rainfall, but vegetation is low due to major run-off and colder temperatures at higher altitude. In contrast, valley floors are warmer, used for agriculture, and irrigated from rivers.

### 3.2. Optimised Hot Spot Analysis of Environmental Variables

Optimised Hot Spot Analysis (OHA) indicated that the optimal scale at which measured values of environmental variables were spatially correlated were between five to six kilometres. Mean values of measured landscape and climatic variables for all analysed nests indicated most nests were found at sites with high rainfall, cooler maximum temperatures and a short distance (~1 km) to the nearest main river system (Table S2). It is important to note that the OHA were conducted on the values measured at the nest and not spatial point itself (e.g., the mean annual rainfall at the nest location).

Given this importance of long-term climatic variables related to moisture and temperature stress for *V. germanica*, OHA maps of these environmental variables were produced for visual analyses and spatial interpretation of the statistical results (Figure 4a,b; Figure S2a–e). From these maps it is evident that there is significant spatial structure in conditions where nests are found. For example, with the ‘distance to main river’ variable, there are distinct clusters (e.g., greater area around Stellenbosch) which are close to rivers (blue spots) while other areas (Paarl and Wellington) nests are generally further from rivers (red spots) (Figure 4a). In comparison, other geographic splits appear in the mean annual rainfall map, where the same neighbouring clusters (i.e., the greater area around Stellenbosch) indicate a split between high values (red spots) occurring next to low values (blue spots) (Figure 4b). Such differences in the distribution of significant hot and cold spots can also be seen in the other environmental variables (Figure S2a–e).

**Figure 5:**
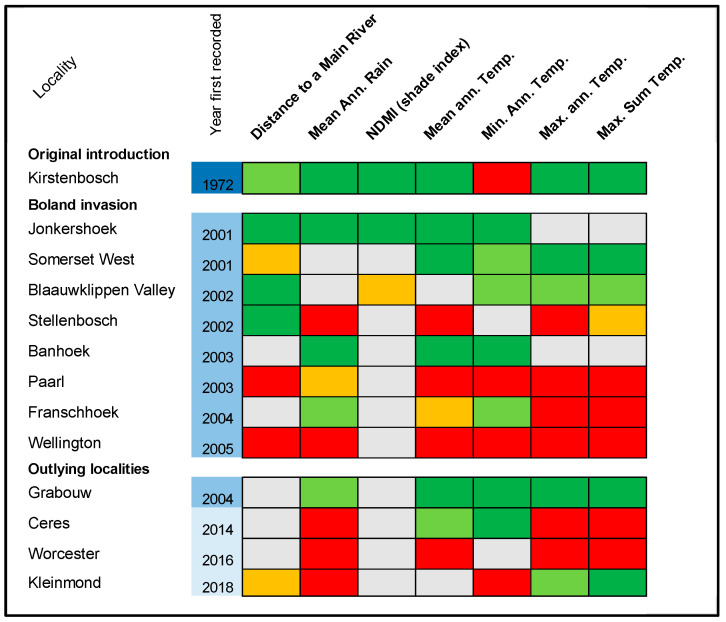
Matrix of spatial clustering (as quantified by Optimised Hot Spot Analysis) of environmental variables relative to localities where *V. germanica* has been recorded. Red blocks indicate significant clusters of unsuitable conditions (at the 99% confidence level; orange at 95%), while green blocks indicate significant clusters of suitable conditions (99% confidence level; light green at 95%), and grey blocks indicate non-significant clustering. [Localities not shown on maps: Kirstenbosch—area between Cape Town and Wynberg; Somerset West—area North of Strand; Blaauwklippen Valley—area South of Stellenbosch; Banhoek—area North-east from Stellenbosch in the direction of Franschhoek].

As it is difficult to simultaneously interpret hot spot patterns for multiple environmental variables, and because clusters of high values for some environmental variables will indicate areas ideal for *V. germanica* (e.g., mean annual rainfall) while for other variables high values will be marginal (e.g., distance to river, mean annual temperature), these spatial patterns were summarised in a locality-environmental variable matrix (Figure 5). Whether conditions were significantly marginal or suitable at localities (and their immediate surrounds) were based on the value ranges of nest records for environmental variables relative to those in the area of absence (Figure 3). Accordingly, the suitability of localities can be gauged across all investigated environmental variables. For example, at Kirstenbosch, where the original invasion was found, all variables (except mean minimum temperature) showed significant clustering in values that represent suitable conditions, strongly suggesting this as very favourable habitat for *V. germanica* (Figure 5). In the second stage of the invasion (post-2000), following the sequence of year first recorded, localities directly bordering the Boland Mountain Complex (Jonkershoek, Banhoek, Grabouw) are also generally suitable habitat for the species, but the habitat becomes progressively less suitable (Franschhoek, Stellenbosch), to marginal when moving towards low lying areas further away from the mountains (Paarl, Wellington).

The chronological order of the invasion shows that *V. germanica* has gradually expanded its range into more marginal landscape habitats over time (Figure 5). Consequently, neighbourhood clustering of hot and cold spots follows a logical structure of core areas located in mountains compared to sinks of less suitable habitats found farther from the mountainous areas (Figure 4a,b; Figure S2a–e).

Three new *V. germanica* invasions were recorded since 2014, two of which are situated directly next to a main road transport network through outlying towns (Figure 1). These localities also radiate from West of the Boland mountains to the North, East, and South. Notably all three these sites are marginal in environmental conditions when compared to Grabouw, which was colonized much earlier (Figure 5).

## 4. Discussion

The results show that nests of *V. germanica* have a predictable non-random spatial distribution relative to the available environmental conditions in the Western Cape Province of South Africa. It is also apparent that there is fine scale spatial structure seen in environmental variables related to moisture stress (function of temperature and moisture availability) measured for nest records, which divide its geographic range into suitable core areas that are bordered by more marginal areas. These geographic limits in available climatically suitable habitat is a likely explanation for the slow invasion in South Africa. This is a significant improvement in quantifying suitable habitat conditions for this invasive species compared to a recent CLIMEX predictive modelling exercise, which broadly described the entire current distribution as optimal to highly suitable [11].

In the past the invasion of this species has been documented anecdotally [36,38]. For decades *V. germanica* was restricted to the Cape Peninsula followed by jump dispersal event to the Boland region after 30 years (although it is only 1-h drive or 50 km). The area in between these two regions is 500 km^2^ with a population of roughly 2.5 million people, but despite this only five nests over the study period have ever been recorded here, indicating first that *V. germanica* nests are not likely to be found in such urban areas and that the invasion from Table Mountain to the Boland represents a discrete jump dispersal event. After the jump dispersal event, it proceeded to spread more rapidly to similar neighbouring farming areas [39]. However, we find that this species is still largely limited to two relatively small geographic areas, namely the eastern parts of Table Mountain (±17 km^2^) and the western side of the two major mountain ranges (the Boland, and Riviersonderend Mountain Complex) (±930 km^2^).

Localised range predictions have not been possible [11] and grid-like monitoring has been hampered by limited artificial bait effectiveness [47]. Consequently, management actions for the control of this species have not been able to be coordinated by government. However, the fact that *V. germanica* is still restricted to a limited geographic area, the coordinated management of this species remains a possibility [39]. More importantly, if the suitable habitat for invasion can be identified from available remotely sensed data, monitoring and clearing will be much easier to coordinate [48]. The significant patterns in the selection of nesting sites in cooler and/or moister landscape conditions than available across the region is suggestive of *V. germanica* queens either selecting specific nesting sites or only nests initiated in these types of habitats can survive to reproduce. Consequently, future queens produced in these areas are more likely to survive. These results will have implications for management of *V. germanica* populations in South Africa, as well as other parts of the world where they are invasive, particularly when in marginal habitats.

Previous work in Australia [13] found that rainfall was correlated with wasp densities, and that sudden ‘wasp outbreaks’ could be attributed to preceding high rainfall events. Horwood et al. [13] also suggested that available water (such as water impoundments and residential water points) could be a major constraint on *V. germanica* invasions and can explain why wasps mostly occur in urban centres where these occur instead of natural habitats. Water is essential for nest construction, maintenance, and to cool and maintain optimal temperature for the brood in the nest, particularly under high temperatures [32]. It is likely that a similar explanation holds for the current observed invasion in South Africa. For example, we found that in some of the hotter areas in its distribution range, wasp nests were mostly well within 1 km (a queen’s average dispersal range; see [49]) from rivers. The proximity to such ideal habitat would facilitate the founding of new nests when new queens can spread from their natal nests. This may imply that river systems provide a corridor for expansion of their range (e.g., [50]) by supplying both shade and water that are important in hotter habitats for wasp dispersal, and further explaining why *V. germanica* is spreading from the edge of mountain ranges into warmer and drier valleys.

Although *V. germanica* is mainly located in temperate regions, D’Adamo et al. [24] described this species as very plastic with regards to high and low temperature conditions, as well as being able to occur in various habitat types. For example, in the Patagonian region of Argentina, extreme frosts result in annual colonies [18]. Under milder climatic conditions, some colonies can become bi-annual or perennial due to lack of cold die-off of the colony in the winter [13]. Currently, *V. germanica* nests in the Western Cape Province of South Africa occur mostly in sunny and arid areas with minimum temperature extremes above 9 °C. This suggests that under such temperate conditions perennial nests should be frequent, but they are in fact rare [39]. *Vespula germanica* is however subject to water stress similar to that experienced in other drier parts in the Southern Hemisphere (e.g., Perth, Australia and Santa Rosa, Argentina; e.g., [11,16,24]), where the absence of frost may be far less important than general moisture availability during summer, in allowing the appearance of perennial nests.

In the Western Cape, a winter rainfall area, there are regular droughts, and the dry season coincides with the seasonal peak in the wasp population [46]. The distribution of successful nests (where a foundress can survive and reproduce new queen daughters) are not random and are located mainly in moist farming valleys or sites with higher moisture availability (rivers and shady habitats, or irrigated orchards); conditions that are also favourable for growing Northern Hemisphere crops in the Western Cape. Our results strongly indicate and support previous findings [11,13,24] that moisture stress is a major constraint on the invasive spread of *V. germanica* populations. Worryingly, *V. germanica* is just one of several potentially invasive social wasp species to South Africa. It is thus important to understand how social wasp invasion progresses in lower moisture areas. Another social Vespid, *Vespa velutina nigrithorax*, are more likely to survive and invade novel environments that have higher precipitation during the driest months of the year [18]. Interestingly, Villemant et al. [18] observed that the CLIMEX predicted distribution of *Vespa velutina* is remarkably similar to that of *V. germanica*. One could thus use the spatial modelling approach we use here for *V. germanica*, to generate finer scale predictions of *V. velutina*’s invasive potential in South Africa, if it were to invade in future.

Apart from natural spread, accidental human transport of mated queens is another important factor in explaining *V. germanica*’s current distribution in South Africa and globally [16,19,33,51]. We found several outward populations (from the original point of introduction), which strongly suggests human mediated dispersal, most probably via road transport (especially likely due to the association with main transport network roads; out of four such populations, three were directly on a major road route). We hypothesise that inseminated queens (the only way of founding new nests) are being transported and deposited into these geographically localised, but still climatically favourable locations. This transportation either happens when the queens are in a hibernating state during winter months, or after mating in the autumn before the onset of winter or early spring when inseminated queens are emerging from winter diapause. The ease by which non-hibernating *V. vulgaris* queens can be transported by car has been documented with several individuals transported in a single event [52]. Traveling time by car from Table Mountain to Stellenbosch is less than an hour, and from Stellenbosch to Ceres, Grabouw or Kleinmond is less than two hours (with vehicles likely not to stop before reaching their end destinations). It is thus highly feasible for non-hibernating inseminated queens to be dispersed in this way (e.g., if they enter cars or busses used for tourist travel) in South Africa. Many queens are potentially being transported in this way, but the chance of being released into a good habitat and surviving to find a nest are slim. Future studies could calculate the probability of human mediated jump dispersal of queens in our study area.

## 5. Conclusions

Due to the strong preference of nesting sites close to river courses, for higher rainfall conditions, medium to-medium-high NDMI values, lower mean annual average, or minimum and maximum temperatures, it is unlikely that *V. germanica* will be able to spread uniformly in natural, unmodified environments. Their impact on natural Mediterranean ecosystems will thus be limited (i.e., Fynbos [Mediterranean] vegetation) unless bordered by human land uses. The close association with human settlements (which aid invasion by providing additional food resources and water [28,53]) means that *V. germanica* will persist and likely increase in density where it occurs in suitable habitats in close proximity to human habitation. This increases the likelihood of accidental transport of mated queens, which in turn increases the chances of jump dispersal. The strong association with human-modified habitats means that where queens select habitats with ideal conditions in South Africa, they are likely to result in negative interactions with humans, which can lead to substantial localised socio-economic impacts [19,54,55]. Although human habitat modification (e.g., irrigated agriculture; [11,39]) likely contributes to the invasion success of *V. germanica* in South Africa, constraints posed by prevailing environmental conditions, limits human mediated invasion of this species.

Furthermore, this study shows that satellite derived climatic and vegetation data could be useful to conduct fine scale searches for possible nesting areas, through mapping and identifying un-surveyed areas. If there is a high degree of overlap between range predictions and observed ranges, these data could be used to predict likely *V. germanica* infestations. Predicting likely invaded habitats more specifically than coarse scale distribution modelling infestation could pave the way for targeted local eradication, which has been advocated as effective for management of these wasps [16,55,56,57]. Consequently, in South Africa and other countries with similar conditions this research assists to better understand the invasion patterns of *V. germanica*.

## Figures and Tables

**Table 1:** Value ranges of environmental variables measured for *V. germanica* nests (median, standard percentiles, and 80% of nest records centred on the median) measured for standard median and quantiles plus additional 10th and 90th percentiles. CV is the coefficient of variation (mean divided by the standard deviation multiplied by 100 to yield a percentage).

	Measures of Variability					
Environmental Variable	10th	25th	Median	75th	90th	CV (%)
Distance to main river course (m)	30.9	117.0	417.1	1007.0	2028.6	118.6
Mean Ann. Rainfall (mm)	693.5	758.8	834.0	896.7	960.2	14.1
NDMI (−100 to 100)	1	9	20	29	37	67.7
Mean Ann. Temp. (°C)	16.43	16.67	16.99	17.12	17.48	2.9
Mean Ann. Min. Temp. (°C)	10.77	10.98	11.23	11.48	12.20	4.9
Mean Ann. Max. Temp. (°C)	21.30	22.26	22.68	22.98	23.55	3.8
Mean Sum. Max. Temp. (°C)	25.61	27.25	28.00	28.25	29.60	5.0

## Data Availability

The data presented in this study are available in Supplementary Table S3.

## Supplementary Materials

The following are available online at https://www.mdpi.com/article/10.3390/insects12080732/s1, Table S1: List of spatial attribute data stored in the GIS and used for analyses [58]. Table S2: Hot Spot analysis summary statistics for neighbourhood analysis of measured climatic and geographic variables. Table S3: Data Availability Statement. Figure S1: Terrestrial area of absence and buffered occurrence of *V. germanica*. Figure S2: Optimised Hot Spot Analysis maps of the environmental variables recorded for *V. germanica* nests records, (**a**) Normalised Difference Moisture Index (NDMI); (**b**) mean annual temperature; (**c**) mean minimum annual temperature; (**d**) mean maximum annual temperature; and (**e**) mean maximum summer annual temperature.
