# Supplementary material for: Spatio–Environmental Analysis of Vespula germanica Nest Records Explains Slow Invasion in South Africa
Source: Insects. 2021 Aug 16;12(8):732. doi: 10.3390/insects12080732 (PMC8396874; doi:10.3390/insects12080732)
Supplement: Supplementary file 1 [file insects-12-00732-s001.zip › sup.pdf]

Ruan Veldtman, Derek Daly and Gerard F.H.v.G. Bekker

Spatio-environmental analysis of *Vespula germanica* nest records explains slow invasion in South Africa

Supplementary Materials: Table S1: List of spatial attribute data stored in the GIS and used for analyses. Table S2: Hot Spot analysis summary statistics for neighbourhood analysis of measured climatic and geographic variables. Figure S1: Terrestrial area of absence and buffered occurrence of *V. germanica*. Figure S2: Optimised Hot Spot Analysis maps of the environmental variables recorded for V. germanica nests records, (a) Normalised Difference Moisture Index (NDMI); (b) mean annual temperature; (c) mean minimum annual temperature; (d) mean maximum annual temperature; and (e) mean maximum summer annual temperature.

Table S1. List of spatial attribute data stored in the GIS and used for analyses.

Description	Source/Reference
Distance to main river courses (m)	Extracted from shape file sourced from http://www.dwaf.gov.za/iwqs/gis_data/river/rivs500k.aspx
Long-term mean annual rainfall (mm)	Van Niekerk and Joubert (2011)
Long-term mean annual temperature (°C)	Van Niekerk and Joubert (2011)
Long-term mean annual minimum temperature (°C)	Van Niekerk and Joubert (2011)
Long-term mean annual maximum temperature (°C)	Van Niekerk and Joubert (2011)
Long-term mean summer maximum temperature (°C)	Van Niekerk and Joubert (2011)
Normalised Difference Moisture Index	Extracted from raster files sourced from https://scihub.copernicus.eu/dhus/#/home

Table S2. Hot Spot analysis summary statistics for neighbourhood analysis of measured climatic and geographic variables. No. of FDR corrected features – meaning that there were multiple test and spatial dependency issues with so many point, but that they have been corrected.

Variable	Optimal distance (m)	No. of FDR[a] corrected features	Mean variable value (± SE)	Min	Max
Distance to main river course (m)	5370	249	769 ± 45	1.6	8699
Mean Ann. Rainfall (mm)	6037.5	275	823.4 ± 5.8	288	1100
NDMI (-100 to 100)	5156	109	0.195 ± 0.006	- 21	50
Mean Ann. Temp. (°C)	6037.5	286	16.94 ± 0.02	14.9	18.7
Mean Ann. Min. Temp. (°C)	5370	260	11.27 ± 0.03	9.5	12.5
Mean Ann. Max. Temp. (°C)	5370	282	22.60 ± 0.04	19.4	24.9
Mean Sum. Max. Temp. (°C)	5370	278	27.84 ± 0.07	23.2	31.3

[a] false discovery rate; [b] nearest neighbour

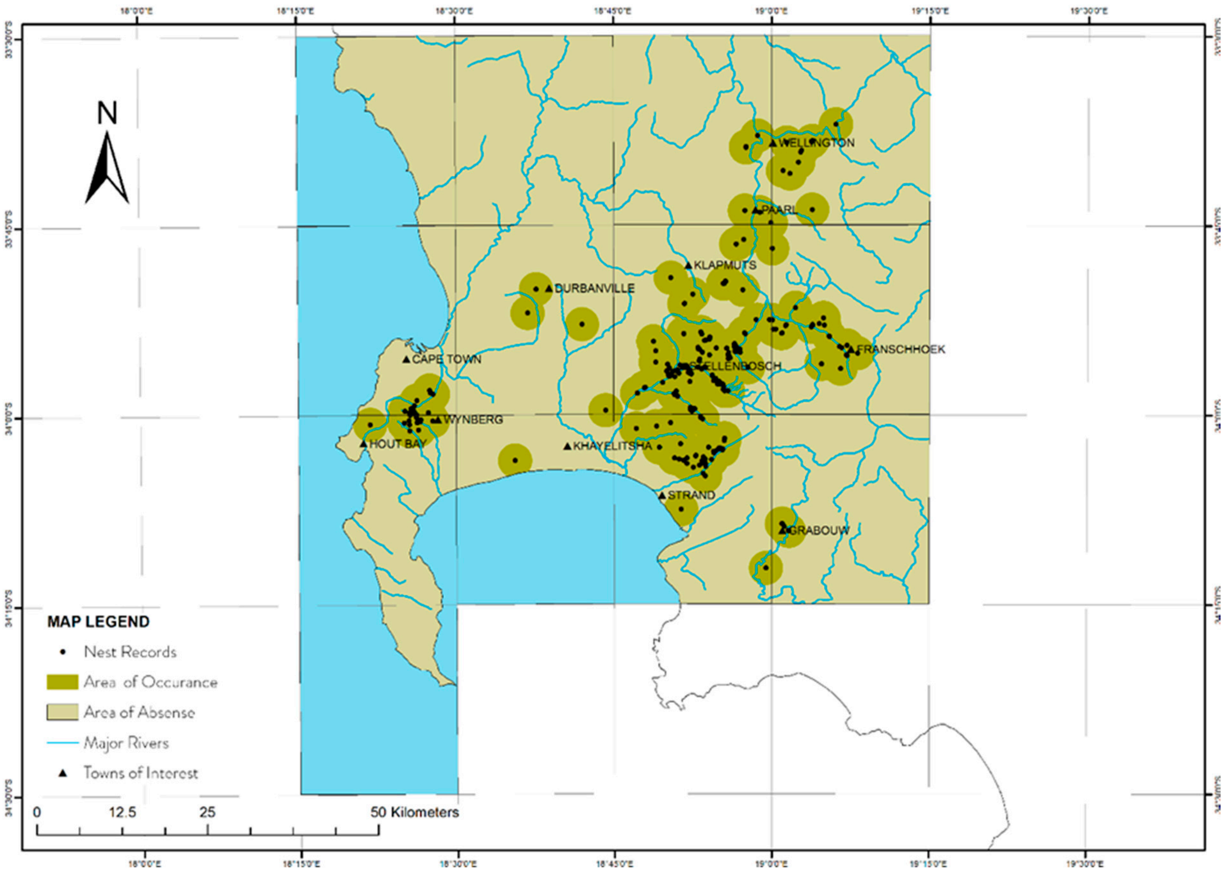

Figure S1: Terrestrial area of absence and buffered occurrence of *V. germanica*. Ten quarter degree cells (15′ by 15′) that contained the 2.5 km buffered area around nest records (taken as its area of occurrence) and the reciprocal area (taken as area of absence) plus cells unoccupied at the same extent from the initial invasion point: Kirstenbosch, Cape Town. Points indicate nests that fell within selected distribution cells (i.e. nest records that indicated jump dispersal were excluded).

(a)

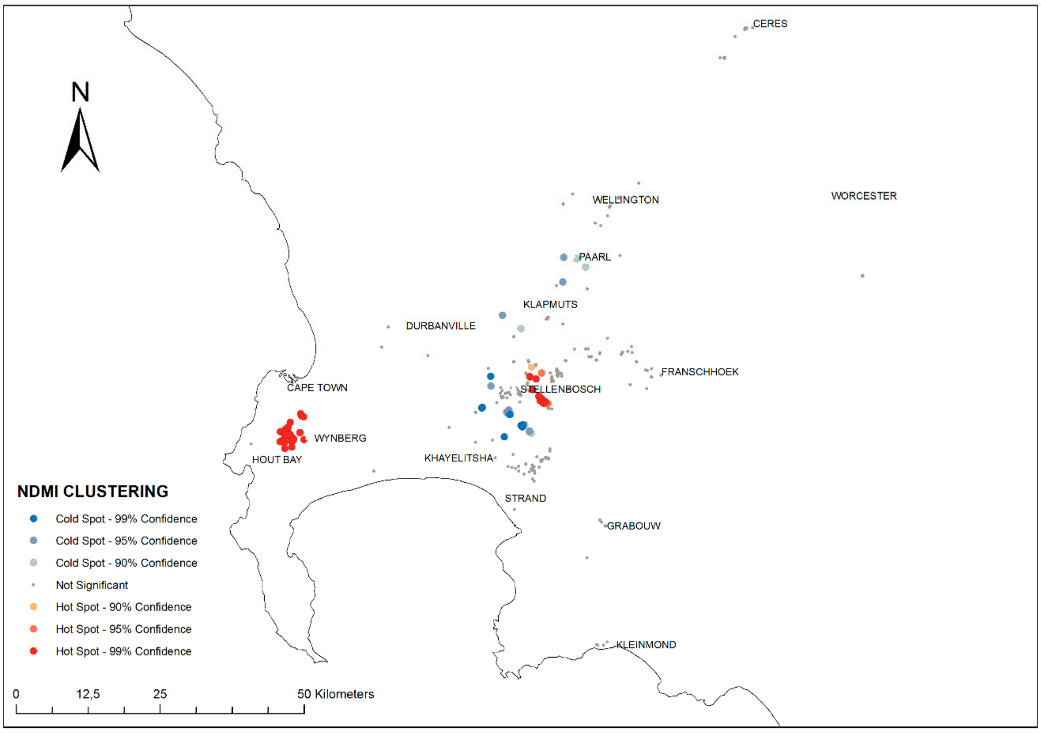
CERES
N
WELLINGTON
WORCESTER
PAARL
KLAPMUTS
DURBANVILLE
FRANSCHHOEK
CAPE TOWN
STELLENBOSCH
WYNBERG
HOUT BAY
KHAYELITSHA
STRAND
GRABOUW
KLEINMOND
NDMI CLUSTERING
Cold Spot - 99% Confidence
Cold Spot - 95% Confidence
Cold Spot - 90% Confidence
Not Significant
Hot Spot - 90% Confidence
Hot Spot - 95% Confidence
Hot Spot - 99% Confidence
0
12,5
25
50 Kilometers

(b)

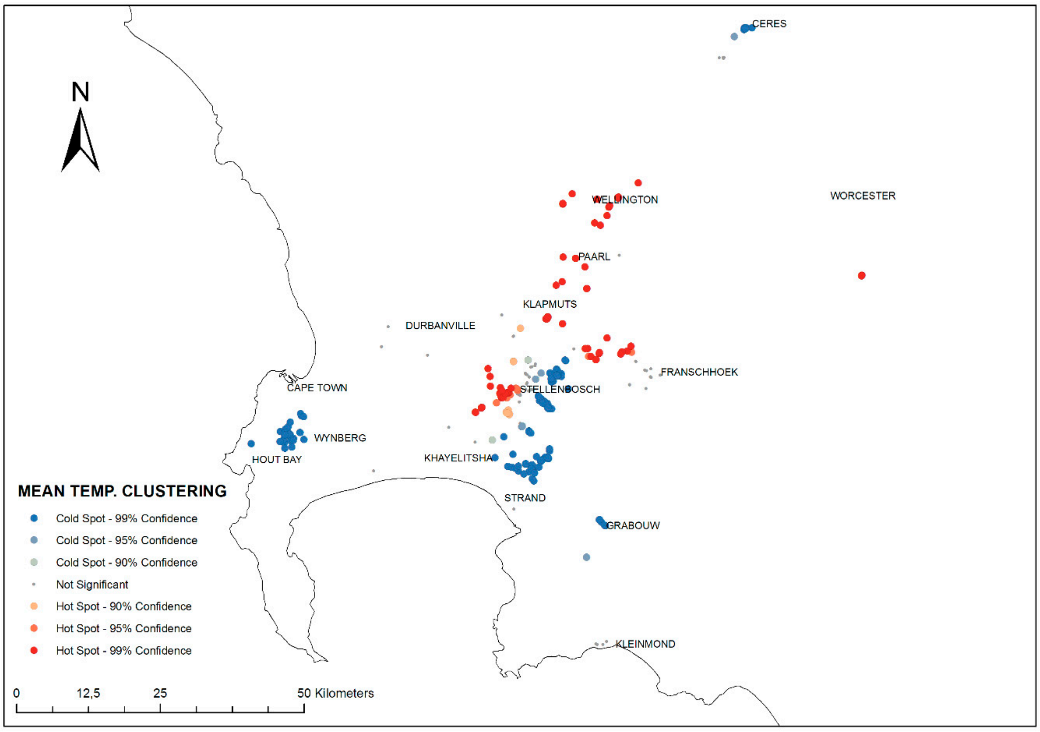
CERES
N
WELLINGTON
WORCESTER
PAARL
KLAPMUTS
DURBANVILLE
FRANSCHHOEK
CAPE TOWN
STELLENBOSCH
WYNBERG
HOUT BAY
KHAYELITSHA
STRAND
GRABOUW
KLEINMOND
MEAN TEMP. CLUSTERING
Cold Spot - 99% Confidence
Cold Spot - 95% Confidence
Cold Spot - 90% Confidence
Not Significant
Hot Spot - 90% Confidence
Hot Spot - 95% Confidence
Hot Spot - 99% Confidence
0
12,5
25
50 Kilometers

(c)

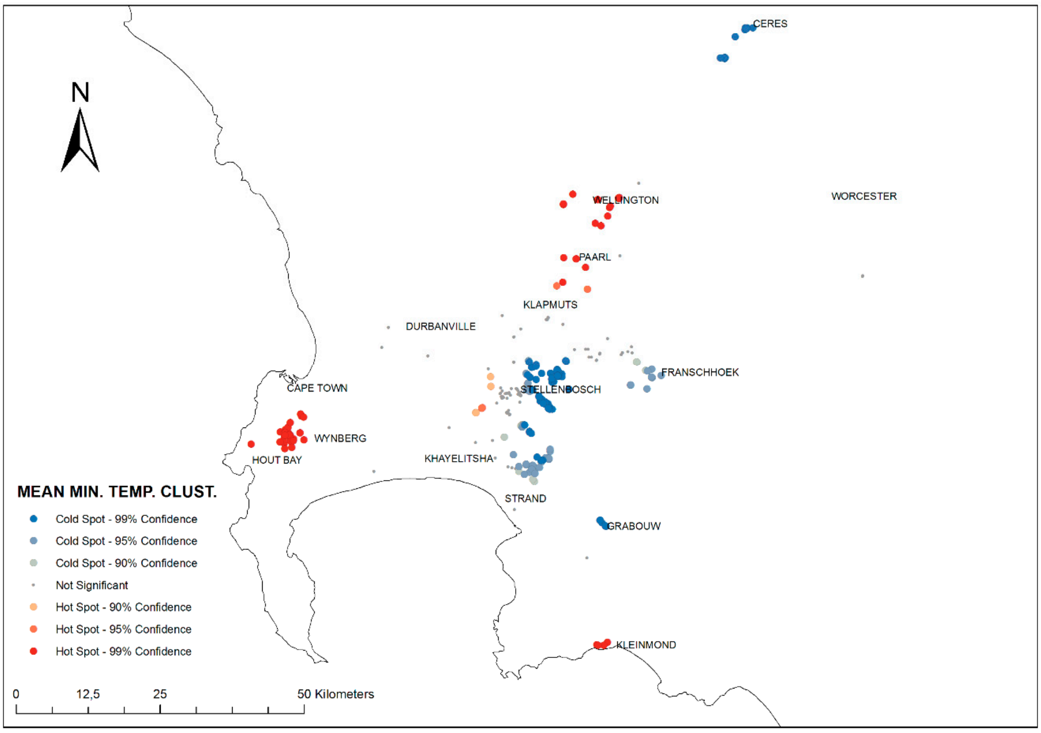
CERES
N
WELLINGTON
WORCESTER
PAARL
KLAPMUTS
DURBANVILLE
FRANSCHHOEK
CAPE TOWN
STELLENBOSCH
WYNBERG
HOUT BAY
KHAYELITSHA
MEAN MIN. TEMP. CLUST.
STRAND
GRABOUW
Cold Spot - 99% Confidence
Cold Spot - 95% Confidence
Cold Spot - 90% Confidence
Not Significant
Hot Spot - 90% Confidence
Hot Spot - 95% Confidence
Hot Spot - 99% Confidence
KLEINMOND
0 12,5 25 50 Kilometers

(d)

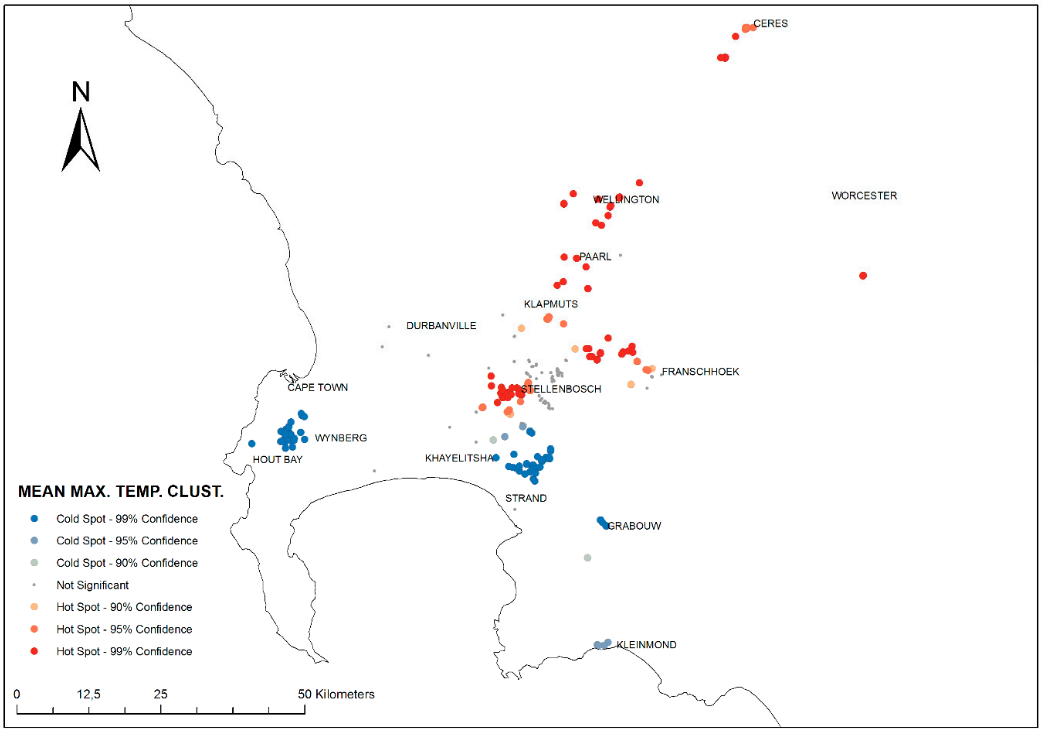
CERES
N
WELLINGTON
WORCESTER
PAARL
KLAPMUTS
DURBANVILLE
FRANSCHHOEK
CAPE TOWN
STELLENBOSCH
WYNBERG
HOUT BAY
KHAYELITSHA
MEAN MAX. TEMP. CLUST.
STRAND
GRABOUW
Cold Spot - 99% Confidence
Cold Spot - 95% Confidence
Cold Spot - 90% Confidence
Not Significant
Hot Spot - 90% Confidence
Hot Spot - 95% Confidence
Hot Spot - 99% Confidence
KLEINMOND
0 12,5 25 50 Kilometers

(e)

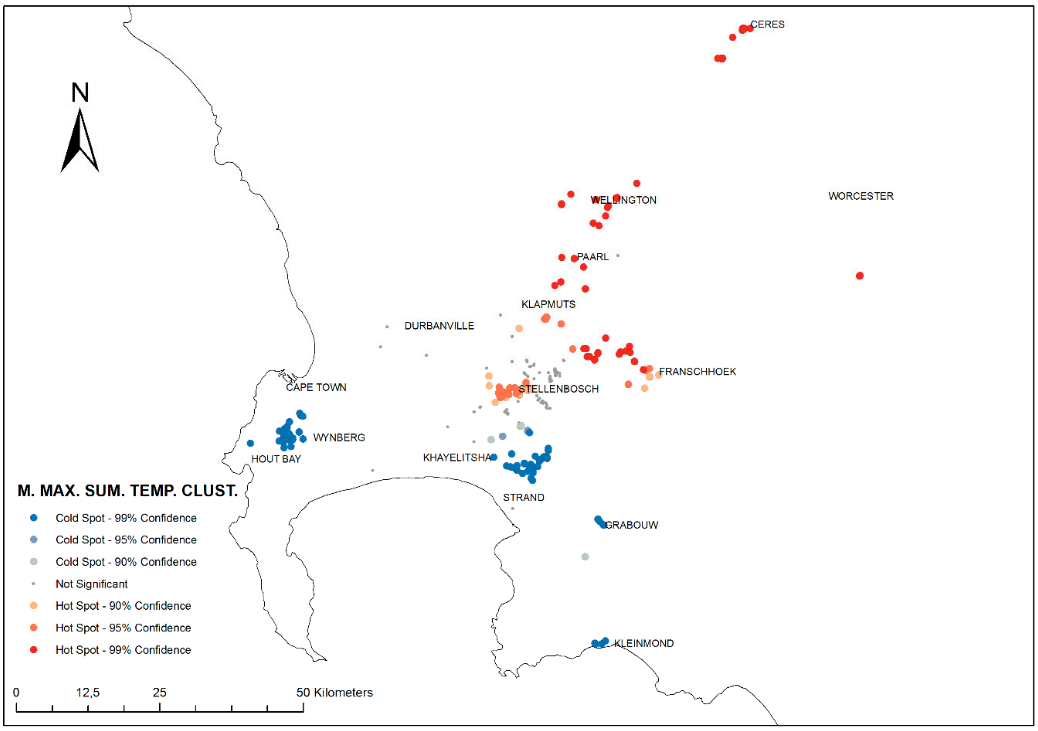

Figure S2: Optimised Hot Spot Analysis maps of the environmental variables recorded for recorded *V. germanica* nests: (**a**) Normalised Difference Moisture Index (NDMI); (**b**) mean annual temperature; (**c**) mean minimum annual temperature; (**d**) mean maximum annual temperature; and (**e**) mean maximum summer annual temperature. A hotspot (red) means significant clustering of high values for nest record data, while a cold spot (blue) shows significant clustering of low values (and not clustering of the nest records themselves).
